# The complete mitochondrial genome of *Homatula pycnolepis* (Teleostei, Cypriniformes) in Lantsang River and its phylogenetic analysis

**DOI:** 10.1080/23802359.2020.1823901

**Published:** 2020-09-29

**Authors:** Rui Li, Xingjian Yue

**Affiliations:** a School of Life Science, Neijiang Normal University, Neijiang, China; b Key Laboratory of Sichuan Province for Fishes Conservation and Utilization in the Upper Reaches of the Yangtze River, Neijiang, China

**Keywords:** Cobitidea, *Homatula pycnolepis*, mitochondrial genome, phylogenetic analysis

## Abstract

*Homatula pycnolepis* is a small-sized benthopelagic fish species in the family Nemacheilidae, superfamily Cobitidea. In this study, the entire mitogenome sequence of the *H. pycnolepis* has been sequenced by using sanger sequencing method. The circular mitochondrial genome was 16,570 bp in length and contained of 13 protein-coding genes, two ribosomal RNA (rRNA) genes, 22 transfer RNA (tRNA) genes, an origin of light-strand replication (OL), and one displacement loop locus (D-loop). The overall base composition was 30.1% A, 26.4% T, 26.8% C, and 16.7% G with 56.6% AT content. Most genes were encoded on the heavy strand except for ND6 and eight tRNA genes. There were 11 regions of gene overlaps totaling 29 bp and 7 intergenic spacer regions totaling 37 bp. Its structure type was similar to the mitogenome of Cobitidea fishes. Phylogenetic analysis with 33 complete mitogenomes of Cobitidea and *2 Macrognathus* fishes in the family of Mastacembelidae suggested that *Homatula* species clustered as one monophyletic clade and *Homatula* was closely related to *Schistura.* Mitogenome information from this study could be a useful basis for conservation and phylogenetics of this species.

In loach genus *Homatula*, there were 16 valid species including 2 new species named in recent years (Gu and Zhang, 2012; Min et al. 2012, 2013; Endruweit 2015; Yang et al. 2017). They are widely distributed in five major drainages in southwestern China (Min et al. 2012). *Homatula pycnolepis* was mainly distributed in the Lantsang River (the upper Mekong drainage) and Nujiang River (the upper Salween River basin) as well as its tributaries (Hu and Zhang 2010). It is a small-sized benthopelagic fish species in the family Nemacheilidae, superfamily Cobitidea, and can be developed as an ornamental fish due to its brightly colored appearance. However, its natural populations declined rapidly in recent years due to overfishing, dam construction in the habitat (Yue et al. 2013; Liu et al. 2016).

Four mitochondrial genomes of 3 *Homatula* fishes have been reported (Shi et al. 2014; Que et al. 2016), but the mitogenome of *H. pycnolepis* has not been reported. Here, complete sequences of mitochondrial DNA of *H. pycnolepis* was determinated by using sanger sequencing method. The samples of *H. pycnolepis* was collected from Jidu River, a littler branch of the Lantsang River, Tue Town, Lanping County, Yunnan Province, China (E99°8′42″, N26°13′55″, 1450 m). The voucher specimens was deposited at Neijiang Normal University (accession number 20080819953).

The total genomic DNA was extracted from the fin tissue using the salt-extracted method (Aljanabi and Martinez 1997) with some modifications. Eighteen pairs of PCR primers were designed to amplify and sequence the complete mitochondrial genome. Four new middle primers were designed for sequencing if entire target segments are not obtained by using the aforementioned primers. The overlapping segments were analyzed using the software Lasergene V11 (DNASTAR) and Mega X (Kumar et al. 2018). The mitgenome was annotated with the MITOS (http://mitos2.bioinf.uni-leipzig.de/index.py) (Bernt et al., 2013), combined with manual corrections.

The *H. pycnolepis* circular mitogenome was 16,570 bp in length and was submitted to GenBank database under accession No. MT783421. It contained 13 protein-coding genes, two ribosomal RNA genes (12s rRNA and 16s rRNA), 22 transfer RNA (tRNA) genes, and 2 main non-coding regions: origin of light-strand replication (OL) and displacement loop locus (D-Loop). The overall base composition of the genome was as follows: A (30.1%), T (26.4%), C (26.8%), and G (16.7%) with a slight AT bias of 56.5%. It was an average AT bias rich feature of teleost mitochondrial genomes.

Its structure type was similar to the mitogenome of Cobitidea fishes (Shi et al. 2014; Ye et al. 2014; Wang et al. 2016; Wei et al. 2016). Most mitochondrial genes were encoded on the heavy strand except for ND6 and eight tRNA genes (tRNA^Gln^, tRNA^Ala^, tRNA^Asn^, tRNA^Cys^, tRNA^Tyr^, tRNA^Ser(UCN)^, tRNA^Glu^, tRNA^Pro^).

The length range of the base of 13 protein-coding genes was 168 bp (ATP8) to 1839 bp (ND5). ATP8 and ND5 of *H. pycnolepis* were 3 bp or15 bp longer than that of cyprinid fishes. The majority of protein coding genes (PCGs) initiated with ATG codon whereas COI gene initiated with GTG codon, which was similar to other fishes of Cobitidea and Cyprinidae (Li et al. 2013; Que et al. 2016; Yue et al. 2016). The termination codons of PCGs were TAA (ND1, COI, ATP8, ATP6, ND4L, ND5), TAG (ND2, ND3, ND6) or an incomplete single T residue (COII, COIII, ND4, cyt b), which was similar to most fishes too.

Twenty-two tRNA genes were interspersed among the rRNA and PCGs, ranged in size from 66 bp (tRNA^Cys^) to 76 bp (tRNA^Lys^). There were two forms of tRNA^Ser^ (UCN and AGY) and two forms of tRNA^Leu^ (UUR and CUN). 12S rRNA (954 bp) and 16S rRNA (1676 bp) were located between tRNA^Phe^ and tRNA^Leu^ and separated by the tRNA^Val^ gene.

There were two major noncoding regions. The origin of light-strand replication, between the tRNA^Asn^ and tRNA^Cys^, was 31 bp fragment and has the potential to fold into a stem-loop secondary structure. The displacement loop locus (control region) was 916 bp sequence located between the tRNA^Pro^ and tRNA^Phe^ genes, with a high AT content of 65.07%. There were lesser AT repeats than Cyprinidae fishes in D-Loop. There were 11 regions of gene overlaps totaling 32 bp (varying from 1 to 13 bp) and 12 intergenic spacer regions totaling 47 bp (varying from 1 to 13 bp).

To confirm the phylogenetic location of *H. pycnolepis* within the superfamily of Cobitidea, Thirty-two complete mitogenomes of Cobitidae were obtained from GenBank, and two *Macrognathus* fishes in the family of Mastacembelidae was used as out-group. Phylogenetic analysis of 35 complete mitogenomes was conducted based on maximum likelihood (ML) analyses implemented in Mega-X (Kumar et al. 2018). Phylogenetic analysis results supported that 4 *Homatula* species (5 sequences) clustered as one monophyletic clade and *Homatula* was closely related to *Schistura* (Figure 1).

**Figure 1.**
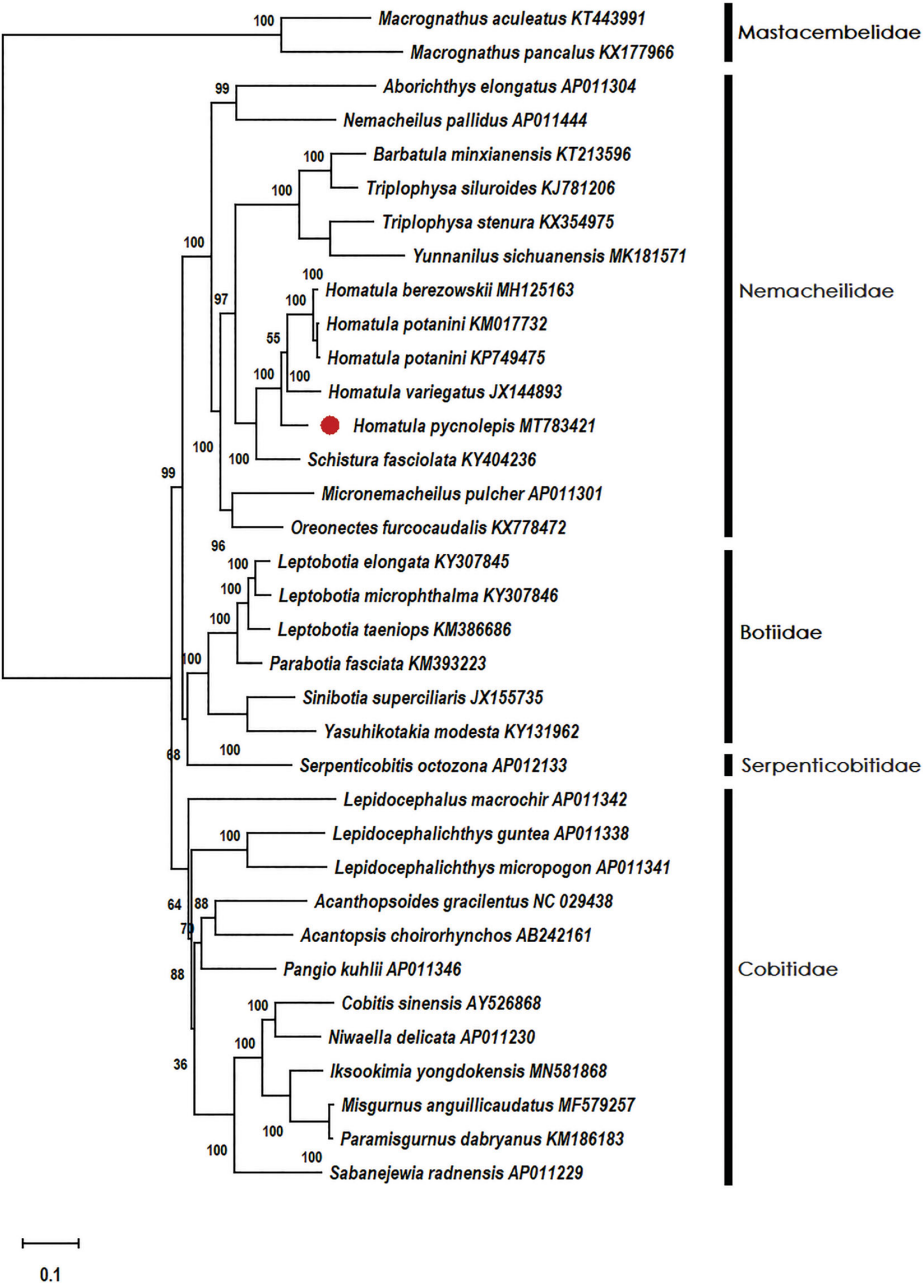
Phylogenetic relationships among 33 complete mito-genomes of Cobitoidea and 2 fishes of *Macrognathus* in the family of Mastacembelidae by maximum likelihood (ML) methods under the G + I nucleotide substitution model. The bootstrap support as computed from 1000 replicates and the bootstrap support values are given at the nodes. *Macrognathus aculeatus* (KT443991) and *Macrognathus pancalus* (KX177966) was used as outgroups. ●*Homatula pycnolepis* (MT783421) was the sample in this study.

The mitogenome sequence data of *H. pycnolepis* presented in this work would provide the fundamental genetic data for inferring phylogenetic relationships of *Homatula*, further study on conservation genetic studies for this species.

## Data Availability

The data that support the findings of this study are openly available in GenBank of NCBI at https://www.ncbi.nlm.nih.gov, reference number MT783421.
